# TextTB: A Mixed Method Pilot Study Evaluating Acceptance, Feasibility, and Exploring Initial Efficacy of a Text Messaging Intervention to Support TB Treatment Adherence

**DOI:** 10.1155/2013/349394

**Published:** 2013-12-12

**Authors:** Sarah Iribarren, Susan Beck, Patricia F. Pearce, Cristina Chirico, Mirta Etchevarria, Daniel Cardinale, Fernando Rubinstein

**Affiliations:** ^1^College of Nursing, University of Utah, 10 South 2000 East, Salt Lake City, UT 84112, USA; ^2^School of Nursing, Loyola University, 6363 Saint Charles Avenue, Stallings Hall, New Orleans, LA 70118, USA; ^3^Region V TB Program, Italia 1750, Florida, Vicente López, CP 1602, Buenos Aires, Argentina; ^4^Institute for Clinical Effectiveness and Healthcare Policy, Dr. Emilio Ravignani 2024, C1414CPT, Argentina

## Abstract

*Objective*. To assess a text messaging intervention to promote tuberculosis (TB) treatment adherence. *Methods*. A mixed-methods pilot study was conducted within a public pulmonary-specialized hospital in Argentina. Patients newly diagnosed with TB who were 18 or older, and had mobile phone access were recruited and randomized to usual care plus either medication calendar (*n* = 19) or text messaging intervention (*n* = 18) for the first two months of treatment. Primary outcomes were feasibility and acceptability; secondary outcomes explored initial efficacy. *Results*. Feasibility was evidenced by high access to mobile phones, familiarity with texting, most phones limited to basic features, a low rate of participant refusal, and many describing suboptimal TB understanding. Acceptability was evidenced by participants indicating feeling cared for, supported, responsible for their treatment, and many self-reporting adherence without a reminder. Participants in the texting group self-reported adherence on average 77% of the days whereas only 53% in calendar group returned diaries. Exploring initial efficacy, microscopy testing was low and treatment outcomes were similar in both groups. *Conclusion*. The texting intervention was well accepted and feasible with greater reporting of adherence using text messaging than the diary. Further evaluation of the texting intervention is warranted.

## 1. Introduction

The mobile phone is cited as the most rapidly adopted technology on the planet [1]. The figure of 6.8 billion mobile phones subscribers in 2013 is nearing the number of people on earth [2]. In an attempt to combat the continued challenges of tuberculosis (TB) as a major global health problem researchers have proposed using short message service (SMS) or text messaging to improve TB treatment adherence [3]. TB treatment adherence remains a challenge in TB control efforts and nonadherence can lead to poor outcomes, such as prolonged infectivity, increase in the risk of relapse after treatment, generation and propagation of drug resistance, treatment failure, and increased mortality [4, 5]. Heightening TB patients' involvement in their own care and improving communication between patients and their healthcare team have been recommended to improve adherence [3, 6].

Argentina has high access to mobile phones and a need to improve TB treatment outcomes. With an estimated population of 40.8 million, there was a reported 59.6 million mobile phones in service and 11.3 billion SMS messages sent in January 2013 [7]. Argentina's National TB Program acknowledges that the overall TB treatment success has varied little and has made no significant improvement over the past 10 years [8]. The World Health Organization (WHO) TB target success rate is 85%; however, in Argentina, the average reported treatment success rate for sputum-smear positive cases was 46% (2008–2010) [9]. Furthermore, about half of the TB cases were reported to have received treatment by self-administration [10].

SMS interventions have been suggested to improve communication and lead to health behavior changes [11, 12], yet few TB researchers have put it to the test. The only text message based TB intervention identified in the literature was a nonrandomized study in India with 30 participants conducted by Mohammed et al. [13] to understand user perceptions, acceptability, and engagement with an SMS system. The intervention involved sending daily text message reminders and asking participants to respond with texting-in the time they took their medication. Mohammed and colleagues report a mean response rate of texting back when patients reported taking their medication of 57% that trended from 62% at the beginning of the trial to 49% towards the end [13]. Therefore, there is limited evidence of applying SMS as the mode of intervention delivery for TB management. Other researchers have tested the video feature of mobile phones to conduct directly observed therapy (DOT) or M-DOT (also reported as V-DOT for video phone) [14–16]. In the US, DeMaio et al. [14] found 95% adherence with six TB patients using V-DOT. In a retrospective cohort study in Australia Wade et al. [16] identified 18.9% (95% CI: 12.2–25.4) (*N* = 128) fewer missed observations than in the traditional DOT using M-DOT and it was calculated to be cost effective. Hoffman et al. [15] evaluated M-DOT in Kenya with 13 patients and their supporters in a proof-of-concept study with 11 completing the 1-month trial; adherence data were not reported. In all of these studies the video capable mobile phones were provided to the participants and almost all participants found the model acceptable. In a third approach, Kunawararak et al. [17] conducted a randomized control study in Thailand with 98 multidrug resistant (MDR) and nondrug resistant TB patients comparing outcomes from daily reminder calls to the patient's mobile phones by a TB center officer to DOT using a family member as the treatment observer. Researchers found higher completion success rates in both groups who received daily calls to their mobile phones (MDR and nondrug resistant TB patients) (*P* < 0.001) and a significantly higher sputum conversion rate in patients with MDR (*P* < 0.001) receiving daily calls compared to the patients in the DOT with a family member observer.

Factors influencing adherence, and ultimately TB treatment success, are varied and may include limited family or healthcare system support, impact on patient's ability to work, lack of knowledge regarding disease and treatment, and social issues related to stigma [18, 19]. The complexity of these factors suggests a multifaceted approach to its treatment management. To improve treatment success and promote treatment adherence, innovative solutions that are readily accessible, convenient, flexible, personalized, and cost effective and that can empower individuals need to be identified and systematically evaluated. The purpose of this presentation is to detail the results of a mixed method evaluation of an interactive multifaceted SMS-based intervention with patients receiving TB by self-administration. In this pilot study we aimed to assess issues of feasibility and acceptability and explore initial results of efficacy of the TextTB intervention to promote adherence to TB treatment. The findings may be applicable to other treatment regimens or in settings with limited resources where self-administration of TB treatment is offered.

## 2. Methods

The study design was a single center, parallel group, mixed methods pilot study. Eligible participants were randomized in a 1 : 1 allocation ratio in blocks of 10 to one of two arms receiving usual care plus either a text messaging based intervention or a medication administration calendar (described below) during the intensive treatment phase, which is the first 2 months of treatment. Primary outcomes were to identify SMS intervention feasibility and acceptability. Secondary outcomes to explore initial efficacy included assessing sputum smear or culture conversion and treatment success.

Two other publications have emanated from this study. The first describes the collaborative development of the text messaging based intervention identifying considerations and decision making rationale along with the theoretical model used to design the educational text messages [20]. The other is a sociotechnical evaluation of the intervention to identify technical considerations to improve the intervention for application to a larger patient population [21].

### 2.1. Informed Consent

This study was approved by the institutional review boards (IRB) from the University of Utah and Hospital Italiano, an independent IRB in Argentina. The primary investigator (PI) provided all participants with a verbal and written description of the study and obtained informed consent in their primary language, Spanish. Participants were compensated for their time in the form of mobile phone credit, virtually added to their mobile phone account at the end of each month (~25 USD equivalent). Participants in the intervention group received extra phone credit to cover the cost of the intervention and for participating in an interview.

### 2.2. Inclusion and Exclusion Criteria

Patients initiating TB treatment who met the inclusion criteria were recruited and enrolled consecutively from November 2011 to June 2012. Initial inclusion criteria included newly diagnosed confirmed TB patients ≥18 years of age who were initiating TB treatment for the first time; had no known TB drug resistance or HIV positive status (HIV status per self-report or medical record); elected to continue treatment and followup at the study site; owned or had access to a mobile phone; and were able to operate the mobile phone to communicate using text messaging. We excluded patients who were severely ill (e.g., requiring hospitalization) or who resided in the same household where another participant was already recruited.

An IRB addendum was approved to expand eligibility criteria after trial commencement to include patients receiving treatment, who had previously completed or had abandoned treatment or had other forms of TB. This change was made because there were many patients being excluded for these reasons (*n* = 15) and the research team felt they might benefit from the intervention.

### 2.3. Study Setting

Participants were recruited from an outpatient clinic within a public pulmonary-specialized reference hospital located within Health Region V in the province of Buenos Aires, Argentina. Health Region V serves a large geographic region with a population of 3.13 million and accounted for about one-third of the TB cases (incidence 49.2/100,000) within the province of Buenos Aires [22]. The regional reference hospital for respiratory pathologies serves urban, suburban, and rural communities.

### 2.4. Usual Care

At this facility patients diagnosed with TB take all of their TB treatment as outpatients from the time of diagnosis, unless their symptoms are severe and hospitalization is recommended by the attending physician. All participants received the routine standard care provided by their healthcare provider at the outpatient clinic. This included routine clinical and laboratory tests and follow-up appointments according to their healthcare provider preferences. Patients received, in general, a month's supply of medication and were asked to return monthly for follow-up evaluations, or if they had any problems patients could schedule appointments prior to their followups.

### 2.5. Interventions

The SMS intervention, developed in collaboration with a multidisciplinary research team and patients, consisted of four components in which patients (a) were instructed to text-in after medication administration (they received reminders if they did not); (b) received confirmation of receipt of notification; (c) were encouraged to text-in questions or concerns; and (d) received twice weekly educational texts which were based on the Information-Motivation-Behavior Skills model [23, 24]. Participants in the intervention arm received verbal and written instructions at recruitment and were asked to send an initial text to test the program's receipt and function. Participants were informed that the intervention was provided within a clinic based system and available during office hours (Monday to Friday) and that emergencies must be directed through standard routes.

Frontline SMS, an open-source software [25], was the text messaging platform used to store, send, and monitor text messages. A basic modem and laptop were used to perform the intervention. Hands-on training and written directions were provided for team members implementing the intervention. A staff member monitored the received messages daily to identify those who had not texted-in and sent out any query texts that were necessary; for example, “We know that you normally take your medication and notify daily but today we have not received notification, any problems?” Two pulmonologists were available for consult via text messaging if a participant had a specific question or concern which the staff member could not answer. A protocol and patient follow-up algorithm developed with the research team was used to guide decision making.

To compare the groups based on self-reported adherence, participants in the control arm were asked to complete a paper-based medication calendar with spaces to indicate if medication was taken and the time taken. Participants were given a demonstration on how to complete the report. Although there are inherent problems with collecting self-reported adherence data, such as overestimation of adherence [26], using a calendar was the most practical method to assess adherence and more closely represented the standard practice. Patients were receiving treatment by self-administration, would return to followup according to provider's request, and could be asked by providers or nurses if they were taking their medication regularly. Another option, such as conducting a pill count, would not have been feasible in this setting.

### 2.6. Primary Outcome Measures

*Feasibility* considerations included identifying the number of potentially eligible participants and the number of patients with cell phones. *Acceptability* focused on assessing how the intervention was used (e.g., self-reported adherence, types of messages sent/received, and average number of reminders sent per patient), identifying patient perceptions of the intervention, and querying patients on how the texting intervention could be optimized. For primary outcomes we collected baseline information on sociodemographics, and mobile phone access, assessed the text messages, and conducted semistructured interviews at the end of the intervention period with a subset of participants in the SMS group.

### 2.7. Secondary Outcome Measures

Sputum smear or culture conversion from positive to negative and the final treatment outcome was collected by reviewing the patient's medical record or outcome reports in the regional TB program records.

### 2.8. Sample Size

We calculated the sample size of at least 17 per group in order to detect a response rate indicating that the intervention warranted further testing based on Schoenfeld's statistical considerations for pilot studies [27]. We used the WHO target treatment success rate of 85% and the average treatment success rate in Argentina 46–65% from 2008–2010 [9] to identify the desired improvement rate. The recommended cutoff (clinical signal) to merit further testing of a pilot tested intervention in a larger trial was a 71% rate of response (successfully completing treatment) in the intervention group [27]. A sample size of 40 was sought to account for attrition and for chi-square analysis requirements [28].

### 2.9. Randomization

Block randomization of 10 was used to ensure balanced representation in the control and intervention arms. The random allocation sequence was generated using a computer-generated randomized list. Opaque envelopes sequentially numbered and sealed were used for treatment allocation concealment. Patients were recruited during medication retrieval at the nurses' station after seeing the healthcare provider. Because of the nature of the intervention blinding of the group allocation could not be conducted for research staff, patients to other patients, or physicians to their patients. However, physicians were not made aware of the group allocation of their patient unless the patient informed them.

### 2.10. Analysis

*Feasibility* issues are reported as descriptive summaries of recruitment data (e.g., number of potential participants excluded due to no access to mobile phone) and descriptive demographic data (e.g., distance to clinic, type of phone access). *Acceptability* was analyzed in a number of ways. Nine in-person semistructured interviews and three partial interviews with structured questions sent via text messaging were conducted. The in-person interviews lasted on average 25 minutes (range 15–47 minutes). Interviews were transcribed verbatim in Spanish by a local, native Spanish speaker [29]. Interviews and field notes were analyzed using descriptive content analysis to assess perceptions of the intervention and patient recommendations of how to optimize the intervention. Focused transcription Spanish-English translation was verified by the PI who is bilingual and by Argentinian site supporter and co-author Dr. Rubinstein. To assess how the intervention was used a database using RedCap [30] was developed and the SMS data were coded for sender, day (e.g., weekday or holiday weekend), number of messages per day, and type of message (e.g., self-reported adherence, question, or reminder). Self-reported adherence rates were calculated as the number of SMS notifications during clinic days of operation (e.g., Monday to Friday) divided by 60 days minus holidays and weekends. Notification sent with or without reminder was also assessed. We conducted a subgroup analysis to explore adherence rates for those who had personal mobile phone access versus shared access. For the control group self-reported adherence was measured by the number of medication administration calendars submitted and the reported number of days indicating medication self-administration.

Secondary outcomes to explore initial efficacy included comparing sputum smear or culture conversion from positive to negative after two months of treatment and the treatment outcomes (e.g., treatment success, abandonment, treatment failure) across groups. Treatment success rate was defined as the percent of patients who either completed treatment (without bacteriological confirmation) or were confirmed cured (negative sputum smear or culture in last month of treatment and on at least one previous occasion) [31].

Statistical analyses were performed using IBM SPSS, version 20 (Chicago, IL). Independent-sample *t*-tests were used for continuous outcomes and chi-square test for dichotomous or categorical variables. Descriptive statistics included means (SD), median, proportions, and ranges. A *P* value less than 0.05 was used to detect a statistically significant difference for all analyses.

## 3. Results

During the recruitment period we enrolled 37 participants; 18 were randomly assigned to the SMS intervention and 19 to usual care with medication calendar for the first two months of treatment. Results from multiple data collection methods are combined to present a summary of issues related to feasibility and acceptability. Conversion rates and treatment outcomes are presented in tables to explore initial results of efficacy as secondary outcomes.

### 3.1. Feasibility of Conducting an SMS-Based Intervention in Study Population

**Figure 1:**
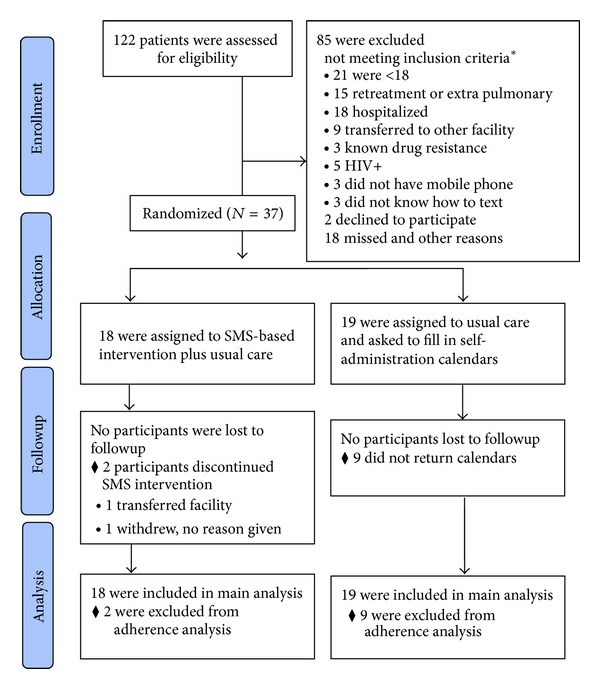
Patient flow diagram *numbers do not sum to 100% because multiple criteria can apply for an individual.

Recruitment data were assessed to identify the feasibility of applying a mobile phone based intervention in this population without supplying a mobile phone to participate. From the 122 patients who were diagnosed with TB during the recruitment period the majority of the patients were excluded due to being younger than 18, being hospitalized for severe TB symptoms, and not meeting initial inclusion criteria (Figure 1). Of those potentially eligible who were assessed for inclusion only three patients were excluded because they did not have a mobile phone or have regular access to a mobile phone, three did not know how to send text messages (one was illiterate), and two declined participation (one was too busy and another indicated that she only wanted to use the calendar if she participated). Patients could be excluded for multiple criteria (e.g., did not know how to send text messages due to illiteracy, multidrug resistant TB diagnosis); therefore the total number of inclusion criteria not met does not sum to the total number of potential participants excluded. Two patients withdrew from the intervention group after random allocation. One required transfer of care to a local healthcare center where DOT was being provided because she could not afford travel cost to hospital outpatient clinic and was suffering from severe TB drug side effects and the other did not provide an explanation for withdrawal after the first day.

Demographic and baseline data were assessed to identify intervention feasibility issues such as having a shared mobile phone, the distance traveled to clinic to identify if DOT would be an option for these patients, and to identify baseline patient understanding of TB. There were no significant differences between the groups by baseline characteristics and demographic variables (Table 1). The average age of participants was 34.43 years with a range of 18–77, and 56.8% were females. Most participants (*n* = 28, 75.7%) had individual (not shared) mobile phones and more had pay-as-you go, without contract mobile phone service (59.5%) and basic mobile phone features without internet (70%). There were 38% of the participants who indicated that they did not or did not always have enough income to cover basic needs (e.g., food and housing). The average travel time to clinic was reported as 66.5 minutes (ranging from 2 to 120 minutes).

Over half of the participants (*n* = 22, 57%) reported that they were not well informed about the disease or treatment upon diagnosis or at their first appointment. When asked to describe what they knew about TB some participants reported knowing very little. Statements included, “zero,” “nothing,” “little to nothing,” “very little,” and “the doctor told me to “take this” and nothing more.” Two participants stated that they thought they contracted TB because, “I was drinking cold drinks, coca-cola” and “I ate out of the garbage when I was little.” Others indicated that TB was “contagious,” “contagious by cough and bacteria come out,” “need to keep house open,” “I have to take medication for a long time,” and “long treatment but curable.”

Overall the participants interviewed indicated that they felt “accompanied,” “cared for,” “had a friend when all others wanted nothing to do with them,” and felt “responsible for my treatment” by texting-in daily. Common themes were being thankful for the support and valuing having someone available to answer their questions. Two participants discussed not forgetting to take the medication because they knew that someone would be checking. One stated “It's like alone it is very complicated there are many pills, a long time…if you do not have someone following you it is likely you will fail treatment.” Three indicated that they would rather send messages daily than go to a clinic daily to receive medication. One patient stated “the experience for me was splendid,” while another indicated that she felt that since this was a study that the research team was “interested in the numbers.” When participants were asked to rank if they would recommend the intervention for other patients diagnosed with TB, 9 of 12 highly recommend, 2 had a low-moderate recommendation, and 1 participant did not text back a response to this question. Examples of text messages received during the intervention indicating acceptance included “thank you for being there,” “thank you for the information,” and “thank you for responding to my question.”

#### 3.1.1. Most Helpful Component

During the in-person interviews, participants described the educational text messages as helpful. One participant indicated that she shared the educational text messages with family and friends to help educate them on the disease. When asked what component of the intervention was most helpful 9 of 12 indicated *all* intervention components were helpful. The other three participants interviewed reported the most helpful intervention components as notifying daily and being able to consult; educational messages and reminders; and educational messages. During the intervention some participants responded to educational text messages. For example, the educational text message regarding TB stigma received a few text replies from participants. One participant texted back “yes many people are even scared to call” and another wrote “often one does not become aware of a disease until they suffer from it. Then one sees and notes the ignorance that we have regarding it and the degree of discrimination that exists!”

#### 3.1.2. How Intervention Was Used

A total of 1320 text messages were sent and 996 were received during the study period (Table 2). On average there were about three questions (ranging from 0 to 9) and two TB drug side effects (0–11) reported per person during the intervention period. Examples of questions texted-in included “I want to know how to take the pills bc there are 8 of them each day. can I take 1 every hour?,” “Is it normal that urine changes reddish?,” “how is TB contagious, if do not talk or breathe in a room can I still spread it?,” “how many days or wks do I have to use a facemask to prevent spread?,” “my knees hurt, could this be related to tx?,” “can any of the meds cause allergies rashes?,” “at some point will my cough go away?” The most common TB drug side effects reported via text messages are in Table 3. The intervention was also used by patients to report going to their appointments, the results of tests of family members, and problems with their mobile phone.

Confirmatory text messages were sent to participants after a notification was received. Staff also used this opportunity to incorporate a motivational message such as, “keep it up!” within the confirmatory text. In addition to answering questions, the TB program staff utilized the text messaging system to notify participants when medication was available to pick up. During the study period there was an unexpected regional and country wide shortage of TB medication. At one point, there was no Ethambutol or Rifampin in liquid form, or some of the second line medications. The shortage began December 1, 2012, and the last text message (*n* = 6), which indicated that Ethambutol had arrived and could be picked up, was sent on March 20, 2013. Because of the shortage patients were allocated medication for shorter periods, for example, 5 days or 2 weeks depending on availability to make the medication last. Patients were informed to keep checking in for when the missing medication would arrive. This unexpected event likely had an impact on the pilot study.

**Figure 2:**
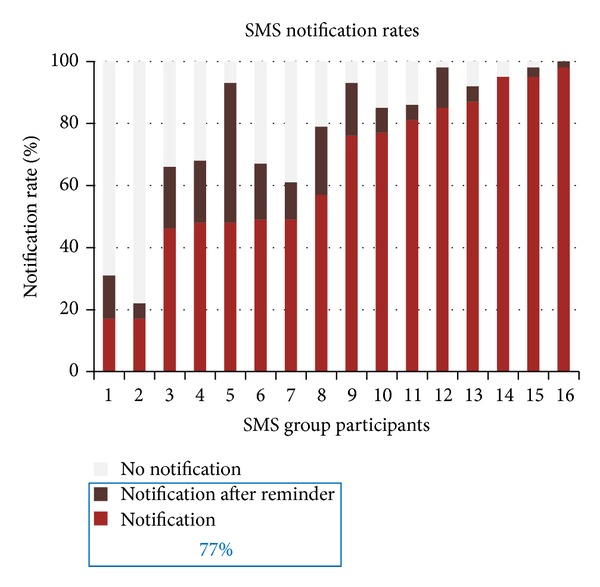
SMS notification rates in access to mobile phone.

The mean notification rate (texting-in) of the SMS group was 77% (SD 23.5, range 22–100) (Figure 2). Of the notifications (*n* = 407), 83% were sent without a reminder. In the SMS group one participant notified the team (texted-in) that she was not taking medications because they were on hold by provider for a week due to having TB drug side effects from the medication.

During the interviews three participants indicated that they had family members or significant others text-in on their behalf. The participants who notified less than 50% of the time were two of the three who shared a phone. These two participants reported a change in access to the mobile phone. For one the shared phone was stolen while the other indicated that his relationship with his girlfriend, with whom he shared the phone, changed and he no longer had access to a phone. The latter obtained a personal mobile phone and reinitiated notifying staff.

Reasons for not notifying were texted-in by some participants and included texting-in the next day “Sorry I did not notify my cell phone fell but I fixed it do not worry I took my meds thank you for caring I say it from my heart greetings to all,” another “Yes I am continuing to take my medication I did not respond bc my phone got wet and I had to put chip in another phone.” Two texted, “if I do not send msg means that I have ran out of credit” and “I took the meds forgot to send msg” and others texted-in that the mobile phone battery ran out during an extended power outage or that they were out of town without mobile phone reception. During the interview one stated “I know it is me that lacks motivation to send message daily” and recommended notifying less often or calling to check on her rather than asking her to text-in daily.

Although participants were not required to do so, some texted-in on weekends or on the next workday after a weekend or holiday, notifying that they had taken their medication for the prior days (e.g., “took medication today and Saturday and Sunday as well”) (mean 4.3 text messages SD 3.3, 0–10, with on average 8 weekends over 60 day period).

Of the participants in the control group, 12 (63%) and 10 (53%) returned calendars for the first and second months, respectively. Therefore, the self-reported adherence rates could not be compared between the groups due to missing information. Of those who did return the calendar there was a 100% self-reported adherence. On the calendars one participant reported going to the emergency room due to fever and another indicated that he did not take Ethambutol because it was not available for 2 days due to the medication shortage.

#### 3.1.3. Recommendations of How to Optimize the Texting Intervention

Recommendations from those interviewed to optimize the texting intervention included continuing the intervention for the full course of treatment; providing more information, including messages from other patients; possibly texting-in less often, for example, once per week; having option to email messages; and offering in-person consultation in addition to text messaging intervention when patients come to clinic to pick up medication. One participant who inconsistently texted-in, partly due to technical/modem problems, stated “might be better to call to see how the person is doing.”

### 3.2. Initial Efficacy

#### 3.2.1. Conversion Rates

Follow-up microscopy testing after two months of treatment was low with only 15 (41%) participants having a follow-up test reported (Table 4). Because of the low rate tests of significance could not be conducted, but it appears that there was no difference.

#### 3.2.2. Treatment Outcomes

Treatment success was high in both groups with 34/37 (92%) completing treatment successfully (Table 5). Two (5%) participants abandoned treatment, one was diagnosed with a nontuberculous mycobacterium after being treated for TB, and no participants failed treatment or died from TB. There were 5 participants who were reported to have transferred to another healthcare facility, but treatment outcomes were documented in their medical record. At the point of final treatment collection, one participant in the SMS group was reported to have abandoned treatment. However, this participant had continued to notify (text-in) after the documented abandonment date, and during the exit interview this participant reported transferring care to a local healthcare facility; however this was not documented in her medical record.

## 4. Discussion

The investigators in this study assessed issues of feasibility and patient acceptability of an SMS-based intervention and explored outcomes of initial efficacy. The multiple component interactive intervention was developed as a comprehensive package in a low-resource setting in which patients routinely receive TB treatment by self-administration. To our knowledge this is the first study with TB patients to trial a mobile phone-based intervention, which goes beyond reminders and may be applicable for resource limited setting where video phone access is not yet commonly accessible and DOT is not offered. Our findings suggest the TextTB intervention was feasible to implement in this population and well accepted as demonstrated by patients' statements of approval, their use of the system to ask questions, report TB drug side effects, and report self-administration. Further, there was greater reporting of adherence in the SMS group than with the use of a medication diary with low numbers of patients returning their calendars.

### 4.1. Feasibility

From the patients diagnosed during the recruitment period meeting inclusion criteria access to mobile phones was high and most knew how to use the SMS feature. One patient lived alone and did not have anyone close to share a phone with and another had recently had her mobile phone stolen and did not anticipate replacing it immediately. Those without mobile phones indicated that they were willing to participate if they had a phone or a phone was provided. Older age did not appear to be a limiting factor to participate. There was also a low refusal rate, which demonstrates potential feasibility of conducting a larger study. Those who consented to participate seemed interested in the texting intervention and eager to have support, which may be a potential reason for low refusal rates. One potential participant who refused indicated that she did so because she did not have time to discuss the study; another indicated that she did not know how to text and was not interested in learning for the study. There were two patients who were angry because of the delay in their TB diagnosis and the clinic nurses did not want to approach them about the study, and another potential patient wanted to go home and think about it and did not return; therefore, they were not invited to participate. Additional potential patients were missed to invite to participate due to other reasons such as, a physician often picking up medication instead of sending the patient, new nursing staff filling in for a sick call from participating nurses, or due to nursing staff not recognizing a new patient during a busy day.

Of the newly diagnosed TB cases identified during the recruitment period the largest group of people who were excluded were those under eighteen years of age. Younger people have been reported to be less compliant with the long treatment [10]. The use of SMS technology as part of everyday communication is high in the younger population, warranting further testing in this age group. In addition, a number of patients were being treated for TB for a second or third time. As previously described, we modified the initial inclusion criteria to be more inclusive of these patients.

Poor access to care and extended travel time has been previously identified as barriers to TB treatment success [32, 33]. In our study patients traveled on average 1 hour and up to 2 in order to be attended to at the regional pulmonary reference hospital. Such distances would likely be prohibitive to provide DOT and in this setting there are no public health nurses who conduct in-home DOT like in other countries such as the United States. However, in Argentina there is a decentralized system with small community healthcare centers dispersed throughout the health region. Anecdotal evidence from discussions with patients points to having more confidence in the larger facilities compared to the smaller clinics, which have varied staff composition and resources. Although the average travel time was substantial, patients indicated that they would rather travel farther for what they perceived was better care provided at larger hospitals compared to the smaller clinics in closer geographic proximity. One of the participants who withdrew from this study had to transfer care to a local healthcare center after 2 weeks in the intervention due to her inability to pay for transportation to the hospital-based clinic for follow-up evaluation for severe TB drug side effects.

Our study supported text messaging as a feasible intervention option. Although access to technology will continue to change, only 30% in this study reported having smart phones or mobile phones with internet access. Therefore, the ability to use the mobile phone video feature, as previously evaluated in other studies [14–16], would have been limited in our population at the time of this study. Our participants were not asked if the video feature would have been an acceptable option for treatment monitoring, but could be considered for future studies.

### 4.2. Acceptability

By evaluating how the text messaging intervention was used and through postintervention interviews we concluded that overall the intervention was accepted by the participants. An aim of the TextTB intervention was to promote a supportive patient-healthcare professional relationship. A strong patient-healthcare professional relationship has been identified as highly important in TB control efforts [34]. The main themes described by participants in the interviews and in the text message were feeling* cared for*, *supported *by staff, and *thankful* for information. Similarly, other researchers using text messaging interventions report patient acceptance and being well received [13, 35, 36]. Most participants indicated that they would highly recommend the intervention to other patients starting TB treatment. Although patients were notified at the end of the 2 months that they no longer were required to text-in daily, some continued to text-in notifications for up to 53 days.

Concerns with health-related information regarding diagnosis being seen by those it was not intended for and associated stigma have been reported as a potential drawback to using mobile phones [6, 13, 35, 37]. Sharing a mobile phone could increase this risk. In our study, however, there was no privacy issues reported and as previously mentioned few researchers have applied mobile phone interventions with patients with TB to identify if this is a barrier. The problems reported by two individuals with shared phones were related to access to the phone rather than concerns with information being received by others. Additionally those with a shared phone reported having a family member or significant other who would send the text message notification on their behalf at times, therefore having others actively participate in their treatment management.

Mohammed and colleagues [13] were the only researchers identified in the literature to have specifically applied text messaging in TB treatment management. Some important differences in our study from that conducted by Mohammed et al. [13] were that our study was a randomized controlled parallel design and the intervention was conducted during the first 2 months of treatment compared to participants who had been on treatment for an average of 3 months. In addition, for our intervention we did not send daily reminders automatically. Patients were asked to notify on their own and received a reminder or inquiry if a notification was not received. TextTB also included educational messages and encouraged patients to text-in questions. Our study results differed in that we had a higher overall reported notification rate and those who shared a mobile phone had the lowest notification rates, in contrast to Mohammed and colleagues, who reported lower response rates for those who owned their own mobile phone. Similar findings included having participants report family members assisted in sending text messages and having participants overall indicate acceptance and being supported by the text messaging intervention. Because our intervention did not automatically send reminders, we were able to detect that many participants notified on their own and used the system to ask questions regarding treatment or what to do after experiencing TB drug side effects. In addition, TextTB provided an opportunity for ongoing education regarding TB treatment.

To enable the comparison of reporting treatment adherence across the groups, the control arm was provided with medication calendars. It was recognized by the research team that adding the calendar was in itself an intervention; however, it was determined to be the least invasive way to compare adherence across groups. However, only about half of the participants in this group returned the calendars and, of those who did, the self-reported adherence rate was 100%, making it hard to compare groups based on this variable. Similarly, in a randomized control trial conducted by Wamalwa and colleagues [38] in Kenya, there were lower self-reported adherence observed in the medication diary group as compared to receiving counseling alone to improve adherence to antiretroviral therapy. In addition, there was a lack of information on how the calendars were completed by the participants, for example, if the data were entered on the calendars daily or at the end of the month. Information on why calendars were not returned was not collected.

### 4.3. Initial Efficacy

Sputum or culture conversion and treatment outcomes were evaluated primarily to assess if patients who were self-reporting adherence were in fact taking their medication as recommended and as measures to explore initial efficacy of the intervention. Patient tracking was paper based and collecting test results and final treatment outcomes was resource intensive and time consuming, highlighting organizational challenges faced by the TB program. Even 1 year after patients initiated treatment, it was challenging for the research staff to collect final treatment outcomes. In our study we found that providers did not consistently request follow-up sputum or culture specimens at the end of the first two months of treatment as recommended by the WHO [39]. Notations in four participants' records indicated that they no longer had a productive cough, in two there were indications of the test ordered but not submitted by the participant, and in the remainder of those without sputum or culture results there was no notation of the test being ordered. Given the low rates of follow-up sputum or culture tests the initial efficacy based on conversion rates could not be adequately assessed. However, using conversion rates as an outcome indicator may be an issue since treatment success is considered either cure with bacteriologic confirmation or treatment completion without bacteriological confirmation [39]. In a study conducted by Lopez et al. [40] assessing the accuracy of follow-up sputum smears to predict treatment outcome, only 56% (*n* = 526) of the population had confirmed conversion.

This trial was not powered to assess the degree to which the intervention contributed to any observable difference nor was evaluating efficacy a primary outcome. However, according to the power analysis for pilot studies which we used to calculate the sample size [27], a treatment success rate of 71% would indicate that further testing of the intervention is warranted. Our final treatment success was 94% in the intervention group. This finding along with the primary findings of feasibility and acceptability supports the testing of this intervention on a larger scale. Yet treatment success was high in both groups. The most recent regional report of treatment outcomes, analyzing data up to 2011, indicated that the regional treatment success was 77.6%. Therefore, both interventions seemed to improve treatment success compared to the latest regional data which may point to any additional support provided being helpful.

### 4.4. Recommendations for Improvement and Issues to Consider for Larger Trial

Reflecting on recommendations from participants, this intervention could be expanded to include other options to make notifying and maintaining contact with healthcare staff more convenient to the patient. For example, having the option to email notifications or questions could be added to the intervention. This, however, could add to the workload of the staff member and possibly be limited due to power outages or loss of internet access. Nonetheless, it could provide both the staff and the patient with a backup option if mobile phone credit runs out or the phone is lost. In addition, participants recommended that the intervention continue for the full course of treatment and include more education.

## 5. Limitations

Although this was a small pilot study rich data were obtained through both qualitative and quantitative methods. Specific factors, applicable to this setting and other resource-limited settings where mobile phone access is high, are highlighted. Nonetheless, this study has some important limitations. First, self-reporting is considered to be subject to systematic biases that are prone to overstating medication regimen adherence [41]. In the intervention group, conducting a daily assessment rather than over a month period may limit the bias of overstating adherence. Because of this potential bias, although not a primary aim, we wanted to assess sputum or culture conversion and treatment outcomes for objective measures to support evidence of actual treatment adherence. Because of the low rate of control group participants returning calendars and the low number of follow-up tests conducted after two months of treatment, we were limited in our ability to compare groups based on self-reported adherence and conversion rates. Additionally, final treatment success rates were high in both groups which may indicate that it is possible that those who agreed to participate were more likely to be adherent with treatment.

Other potential contributing factors to improved outcomes in both groups could include increased visits to the clinic due to the medication shortage and additional education received at the onset of the study. The period during which some TB medication was scarce or not available changed the medication allocation intervals. When medication was available patients standardly received a month supply and when there was a shortage the allocation varied from 3 days to 2 weeks' worth depending on the amount of medication available. Therefore, the amount of contact with the patient and a healthcare team member may have changed during this period.

At recruitment nearly 60% of the participants indicated that they were not well informed by providers on the disease or its treatment and some described inaccurate causes of TB transmission. During recruitment participants were given the opportunity to ask questions, therefore possibly providing an additional educational benefit to both groups. In future studies TB understanding might be better elucidated by asking specific TB knowledge questions at baseline and after intervention to more accurately capture what was learned from educational text messages. In addition, future studies could tailor messages based on baseline knowledge.

## 6. Conclusions

New strategies are needed to improve patient adherence to TB treatment to prevent the spread of disease, worsening drug resistance, and poor treatment outcomes. In settings where self-administered treatment is the usual care early identification of nonadherence to treatment is likely to improve overall treatment outcomes. To empower patients to engage in the management of their own care, promote treatment adherence, and support patients with TB the TextTB mobile phone-based intervention was trialed. Our results suggest that the texting intervention was feasible in this population and overall well-received. Feasibility was evidenced by high access to mobile phones, most knowing how to text, older age not being a limiting factor to participate, a majority of the phones with only basic features, and a low rate of refusal to participate. Acceptability was evidenced by participants stating feeling cared for and supported and utilizing the intervention to meet their needs. Asking participants to text-in after self-administering medication, rather than sending automatic daily reminders, was supported. For some in this study sharing a mobile phone represented a barrier. There was greater self-reporting of adherence using text messaging than with the use of a medication diary. Although final treatment outcomes were similar in both groups feasibility and acceptability results in this study suggest that there is value in assessing the texting intervention in a larger-scale research project. Testing in a younger population and extending for the full course of TB treatment are other research possibilities. Using the texting feature was the most feasible in this population at the time of the study. However, in the near future the patient population may have a greater utilization rate of video or other advanced feature phones. Therefore, being flexible and adapting to quickly changing technology will be required to leverage the benefits that new technologies will provide.

## Figures and Tables

**Table 1:** Demographic and baseline characteristics.

	Control group (*n* = 19)	SMS group (*n* = 18)	*P*
Age (years) (mean, SD, range)	35.05 (17, 18–77)	33.78 (15, 18–75)	0.81
Women	11 (58%)	10 (56%)	0.89
Education (completed)			0.19
Primary	7 (37%)	2 (11%)	
Secondary	10 (53%)	13 (72%)	
Postsecondary	2 (11%)	3 (17%)	
Travel time (min) (mean, SD, range)	63.93 (32.06, 10–120)	69.06 (43.21, 2–120)	0.71
Takes other medications daily	7 (37%)	2 (11%)	0.08
Income covers basic needs (e.g., food)	11 (58%)	12 (67%)	0.58
Mobile phone access			0.29
Personal	13 (68%)	15 (83%)	
Shared	6 (32%)	3 (17%)	
Basic feature mobile phone	16 (84%)	10 (55%)	0.06
Prepaid mobile phone plan	13 (68%)	9 (50%)	0.25
Number of texts sent per day			0.45
<1/day	5 (26%)	3 (17%)	
1–10/day	8 (42%)	10 (56%)	
+10/day	6 (32%)	5 (28%)	
Not sufficiently informed about TB	13 (68%)	8 (44%)	0.37

The number and percent are reported unless otherwise indicated.

**Table 2:** Types of messages sent and received during intervention.

Type of SMS message	*N*	Median	Mean	sd	Range
Text messages received from patients	996	46.00	55.00	33.50	2–131
Questions	46	2.00	2.87	2.55	0–9
Report side effects	32	0	2.00	3.50	0–11
Days continued to notify after intervention	109	3.5	7.79	13.74	0–53
Test messages sent by researchers	1320	77.00	73.00	28.5	18–154
Reminders per patient	170	10.00	9.44	6.59	0–21
Automatic confirmation of notification	307	12.00	17.05	14.11	0–40
Manual confirmation of notification	128	6.5	7.11	6.66	0–21
Educational messages	277	12	15.39	3.42	7–19
Personalized/respond to questions	113	6.5	6.28	4.16	0–14

**Table 3:** Most common TB drug side effects reported in text messages*. *

Side effect	*N*
Stomach problems	10
Rash	4
Muscle ache/pain	3
Low energy	3
Urine color change	2

**Table 4:** Sputum or culture conversion by group after two months of treatment.

Sputum smear or culture	Calendar (*n*)	SMS (*n*)
Remained positive	2	2
Converted to negative	6	5
Not evaluated	11	11

Not evaluated: records indicated no sputum production or no follow-up test ordered.

**Table 5:** Treatment outcomes by group.

Treatment outcome	Calendar (*n*)	SMS (*n*)
Treatment success (cured or completed)	17	17
Failure/continuing treatment	0	0
Death	0	0
Treatment Interrupted/abandoned	1	1
Transferred out/no data	0	0
Other	1	0

Other represented a participant diagnosed with nontuberculous mycobacterium after completing TB treatment for an extended period.
